# Controlling the Charge Density Wave Transition in Single-Layer TiTe_2*x*_Se_2(1–*x*)_ Alloys by Band Gap Engineering

**DOI:** 10.1021/acs.nanolett.3c03776

**Published:** 2023-12-20

**Authors:** Tommaso Antonelli, Akhil Rajan, Matthew D. Watson, Shoresh Soltani, Joe Houghton, Gesa-Roxanne Siemann, Andela Zivanovic, Chiara Bigi, Brendan Edwards, Phil D. C. King

**Affiliations:** SUPA, School of Physics and AstronomyUniversity of St Andrews, St Andrews KY16 9SS, United Kingdom

**Keywords:** 2D materials, transition-metal dichalcogenide, charge density wave, excitonic insulator, angle-resolved photoemission spectroscopy, molecular beam epitaxy

## Abstract

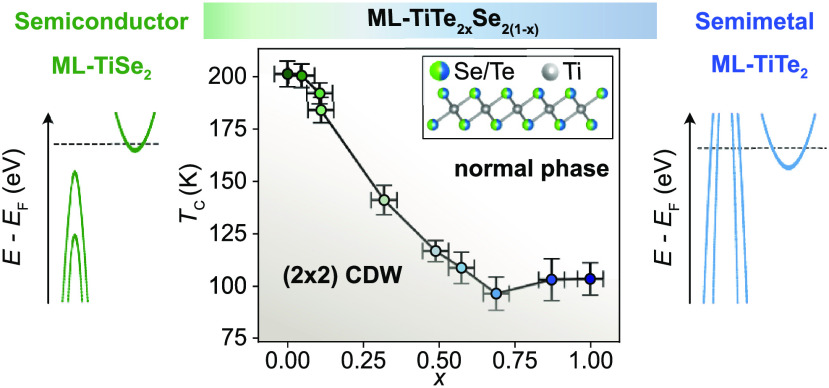

Closing the band gap of a semiconductor into a semimetallic state gives a powerful potential route to tune the electronic energy gains that drive collective phases like charge density waves (CDWs) and excitonic insulator states. We explore this approach for the controversial CDW material monolayer (ML) TiSe_2_ by engineering its narrow band gap to the semimetallic limit of ML-TiTe_2_. Using molecular beam epitaxy, we demonstrate the growth of ML-TiTe_2*x*_Se_2(1–*x*)_ alloys across the entire compositional range and unveil how the (2 × 2) CDW instability evolves through the normal state semiconductor–semimetal transition via *in situ* angle-resolved photoemission spectroscopy. Through model electronic structure calculations, we identify how this tunes the relative strength of excitonic and Peierls-like coupling, demonstrating band gap engineering as a powerful method for controlling the microscopic mechanisms underpinning the formation of collective states in two-dimensional materials.

Numerous collective phases in solids result from an instability of the electronic states close to the chemical potential. The different coupling mechanisms underpinning these instabilities should therefore be highly sensitive to the closing or opening of a band gap at the transition between a semiconducting and semimetallic system. Group IV transition-metal dichalcogenides such as TiX_2_ (X = S, Se, Te) provide an ideal platform in which to explore this, since the energy separation between the Ti 3d derived conduction band and the chalcogenide p-derived valence band strongly depends on the chalcogen present. As the atomic number of the chalcogen is increased along the group from S to Se, the band gap decreases from >0.5 eV in the indirect semiconductor TiS_2_ ^1^ to only 72 meV in bulk TiSe_2_.^2^ This band gap closure promotes the emergence of a (2 × 2 × 2) charge density wave instability in bulk TiSe_2_ at temperatures below 200 K.^3^ When thinned down to the monolayer (ML) limit the CDW instability in TiSe_2_ is preserved, forming an equivalent (2 × 2) CDW phase which is stable up to a slightly higher critical temperature of 220 K.^4,5^

The pairing mechanism underpinning the transition in both bulk and ML TiSe_2_ has been debated for decades, with experimental and theoretical results in favor of either an excitonic or phonon-mediated electron–hole coupling^6−11^ with evidence also for a cooperative role between these two mechanisms.^12−16^ Substituting Se with Te, a semimetallic compound, TiTe_2_ is obtained where both the valence and conduction band cross the Fermi level,^17−19^ as shown schematically in Figure 1a, and where excitonic correlations would be expected to be strongly screened. Surprisingly, a (2 × 2) CDW transition, similar to the one in ML-TiSe_2_, has recently been observed in ML-TiTe_2_, while it is completely suppressed in the multilayer and bulk crystals.^20^ The lower CDW critical temperature (*T*_c_) in the telluride compound points toward a weaker CDW coupling as compared to the selenide case.^5,21^ This suggests that the CDW interaction can be tuned in this system by the chemical substitution of Se with Te, potentially providing a new route to assess the importance of excitonic and lattice contributions to the collective state formation in this system.

**Figure 1:**
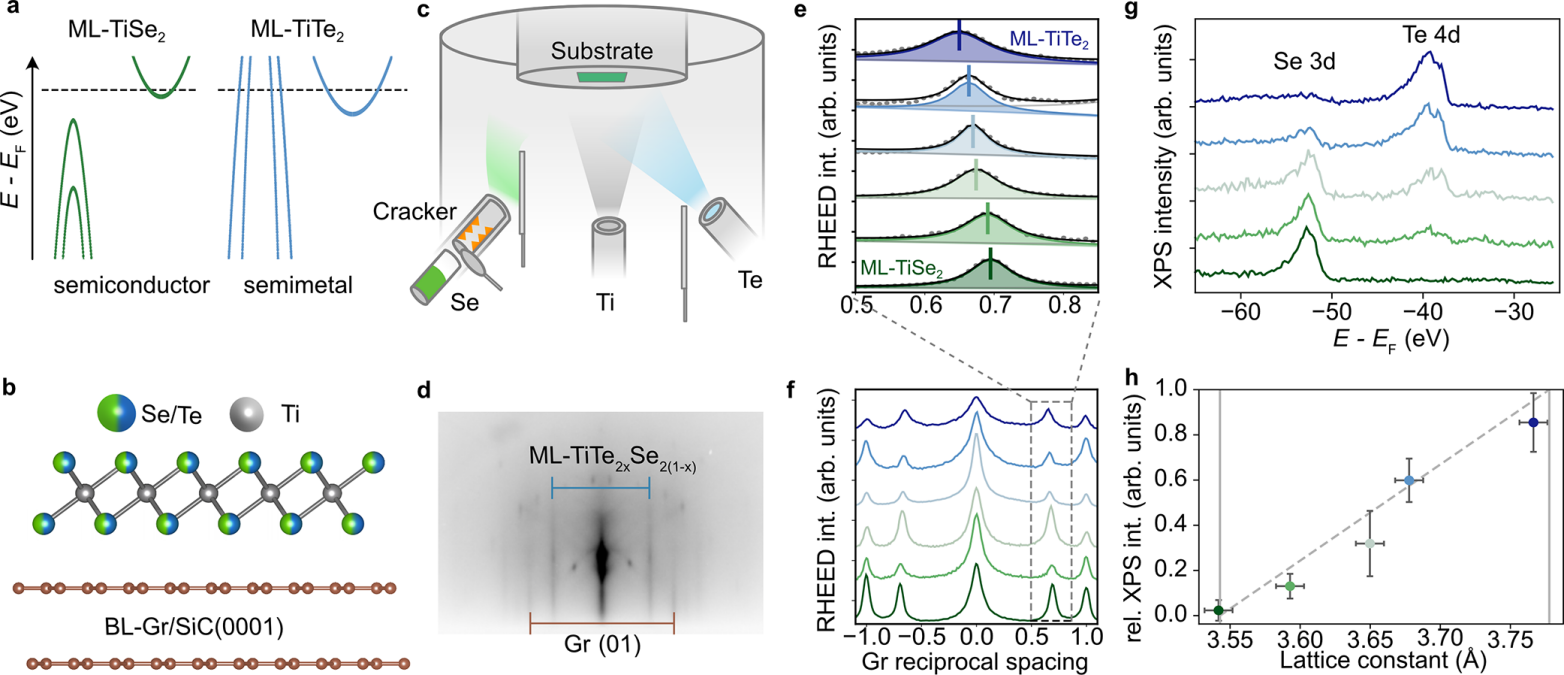
(a) Schematic of the low energy band structure of ML-TiSe_2_ and ML-TiTe_2_. (b) Crystal structure of 1T-ML-TiX_2_ on a bilayer graphene substrate (BL-Gr). (c) Schematic of the MBE system used to grow the ML-TiTe_2*x*_Se_2(1–*x*)_ alloys. Controlling the aperture of the Se cracker valve allows the fine-tuning of the Se atomic flux introduced into the deposition chamber, and therefore of the alloy composition. (d) Typical RHEED image measured after growth showing the diffraction pattern of the alloy film superimposed onto the substrate one. (e, f) By changing the Se/Te flux ratio, the lattice constant of the alloy film changes as evident by the shift of its RHEED pattern. The lattice constant of the as-grown film is measured by fitting cuts of the RHEED images. (g) *In situ* XPS measurement on the as-grown films with different Te content showing the modulation of the Te and Se core level intensities. (h) Correlation between the composition extracted by XPS and the lattice constant estimated from the RHEED images. The dashed line represents the expected linear relation from Vegard’s law.

Here, we report the synthesis of TiTe_2*x*_Se_2(1–*x*)_ monolayers using molecular-beam epitaxy (MBE), and we study the evolution of the CDW instability with alloy composition, *x*. Our *in situ* reflection high-energy electron diffraction (RHEED) and angle-resolved photoemission (ARPES) measurements demonstrate continuous control of the normal state electronic structure with alloy composition. Moreover, our approach allows us to associate changes in the observed *T*_c_ with characteristic features in the normal state electronic structure, in turn pointing to a fine control over the different microscopic mechanisms driving the CDW in this materials system.

ML-TiTe_2*x*_Se_2(1–*x*)_ alloys were grown by MBE on bilayer graphene-terminated SiC wafers using a codeposition of Ti, Se, and Te as illustrated in Figure 1b,c. The substrate was kept at 400–500 °C throughout the growth, as measured by a thermocouple placed behind the substrate. The growth was performed in a highly chalcogen-rich environment (Ti/(Te + Se) flux ratio ≈ 10^3^), for a duration of 70 min following the method outlined in ref (22). Figure 1d shows a typical RHEED image of a film measured after growth, showing the diffraction pattern of both the graphene substrate and the new alloy film. The lattice constant of the TMD layer can be directly extracted by considering the ratio between the reciprocal spacing of the substrate and the grown film. Preliminary growth tests using a chalcogen flux ratio Se/Te = 0.5 resulted in films with a lattice constant equal to pure ML-TiSe_2_ (3.54 Å from ref (23)), suggesting that the Se is much more reactive with Ti as compared to Te. In order to suppress the Se flux, the Se cell shutter was thus kept closed during the deposition with films of different compositions grown using Se fluxes controlled by changing the aperture of the cracker valve of our valved cracker Se source (Figure 1c), allowing for a finer control of the resulting Se partial pressure in the UHV chamber. The Te flux was kept constant for all the samples. Following this method, films with different lattice constants spanning from 3.54 Å (TiSe_2_) to 3.78 Å (TiTe_2_) were grown. This is evident via the horizontal cuts extracted from postgrowth RHEED images shown in Figure 1e,f. The peak position related to the alloy film shifts to lower values relative to the graphene reciprocal spacing (indicating an increase in the film lattice constant) with increasing Te:Se flux during the growth. To determine the resulting lattice constant, the RHEED cuts were fit with five Lorentzian and a quadratic background. The alloy composition was also probed by using *in situ* X-ray photoemission spectroscopy (XPS) measuring the Se 3d and Te 4d core levels, as shown in Figure 1g. The Te content of the film was estimated as
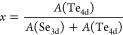
1where *A*(Ch_*n*d_) is the area of the chalcogen core level normalized over the relative cross-section: 0.0314 Mbarn for Se 3d and 0.04979 Mbarn for Te 4d for the utilized photon energy of *hν* = 1486.7 eV.^24^ In Figure 1h we plot the obtained alloy composition from XPS versus the lattice constant extracted from RHEED, showing a clear correlation consistent with Vegard’s law represented by the linear dashed line. This relationship allows us to estimate the film composition directly from the RHEED pattern, providing fast feedback straight after the growth.

To study the normal state band structure evolution across the alloy series, we performed *in situ* ARPES measurements with a photon energy of *h*ν = 21.2 eV at temperatures well above the expected CDW critical temperature. Our measurements are shown in Figure 2c,d. The broadening of the measured spectra increases in the alloy films as compared to the pure end members, reflecting an intrinsic chalcogen site disorder of the alloy 2D crystals. Nonetheless, clear band dispersions are obtained across the series, allowing us to track the alloy-induced changes in the electronic structure. For compositions close to pure TiSe_2_, we observed the top of the two spin–orbit split valence bands at Γ (Figure 2c) shifting toward the Fermi level with increasing Te composition, until the lower valence band maximum disappears above the Fermi cutoff for compositions *x* ≈ 0.41. As the Te content is further increased, the valence band maximum is pushed further up in energy, enlarging the size of the hole pocket at the Fermi level.

**Figure 2:**
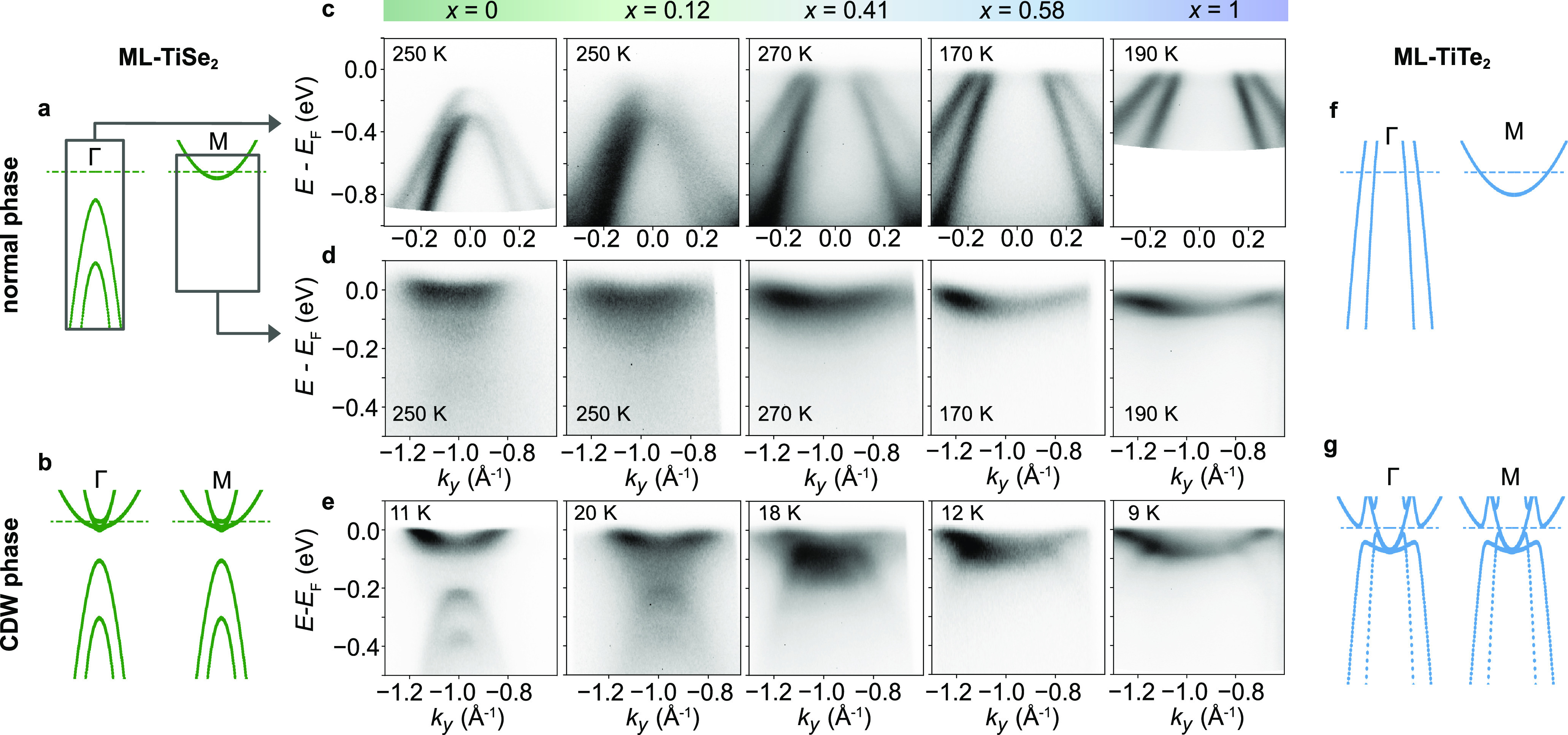
Schematics of the low-energy band dispersion around Γ and M in the normal phase and CDW phase for ML-TiSe_2_ (a, b) and ML-TiTe_2_ (f, g). *In situ* ARPES spectra of ML-TiTe_2*x*_Se_2(1–*x*)_ for different Te contents (*x*) measured (c, d) above *T*_c_ at Γ (c) and M (d), *h*ν = 21.2 eV. (e) Low-temperature measurements at M. The valence replica appears as a consequence of the (2 × 2) CDW transition.

At M, the bottom of the conduction band shows the opposite trend (Figure 2d). We note that the electron pocket in ML-TiSe_2_ already slightly crosses the Fermi level, in contrast to the nominal Ti d^0^ expected electron configuration. This is possibly due to intrinsic charge transfer with the BL-Gr substrate, or due to the presence of residual Se vacancies that act as effective *n*-dopants.^4,5^ Even at temperatures well above *T*_c_, a weak valence band backfolded spectral weight is visible at energies around −0.1 and −0.35 eV, assigned to uncorrelated electron–hole pairs formed by the strong coupling nature of the incipient CDW instability.^5,8^ This effect is still visible in the alloy film at *x* = 0.12, despite the larger broadening of the spectrum. Upon increasing the Te content, the rather small electron pocket becomes larger, with the conduction band minimum moving from *E* – *E*_F_ = −0.03 eV for TiSe_2_ to −0.08 eV in the telluride limit. This continuous modulation of the band structure with the lattice constant indicates the effective synthesis of a homogeneous alloy and demonstrates the control of its composition using our MBE method. The film homogeneity was also tested *ex situ* using small-spot XPS as discussed in detail in the Supporting Information, from which we find only small compositional variations across the grown sample.

To probe how the CDW ground state evolves by changing the Te content, we studied the temperature-dependent evolution of the electronic structure around the M-point (Figure 2e). In both ML-TiSe_2_ and ML-TiTe_2_, a backfolded valence band appears at M, indicating that the system acquired the typical (2 × 2) periodicity of the CDW phase. In Figure 2a,b,f,g, we show schematics of how the orbital selective hybridization between the backfolded valence and conduction bands reshapes the low-energy band structure of ML-TiSe_2_ and ML-TiTe_2_, following the model reported in ref (21). While in TiSe_2_ the hybridization enhances the original band gap, pushing the valence band top away from the Fermi level, in TiTe_2_ the CDW interaction opens new gaps at selected band crossing points, with symmetry-dependent form factors.

In the alloy samples, the characteristic backfolded spectral weight appears at the M point for all intermediate compositions, indicating that the (2 × 2) CDW instability is preserved across the entire alloy series. The sample with *x* = 0.12 shows a backfolded band similar to the one in pure ML-TiSe_2_, with the addition of a broad tail of the conduction band connecting to the valence band top. This anomalous feature might be induced by the complex interplay between CDW ordering and the intrinsic disorder of the alloy lattice. On the other hand, for the Te-rich sample with *x* = 0.58, the spectral signatures appear qualitatively similar to that of the pure Te-based end member. For the sample with a Te content of *x* = 0.41, a clear gap becomes visible at the crossing points between the outer valence band and the electron pocket. We show in Figure 3a how the same orbital-selective band hybridization that describes the CDW transition in the end member compounds reshapes the low-energy band structure at this specific composition. We consider two spin–orbit split valence bands hybridizing with the three backfolded conduction bands at Γ, using the model Hamiltonian approach described in ref (21). Taking an effective hybridization strength of Δ = 42 meV, equal to the one estimated for pure ML-TiTe_2_,^21^ we find that the top of the outer valence band becomes strongly flattened, acquiring a strong Ti 3d character. This explains its relatively strong intensity in the ARPES measurement and indicates that a similar phenomenology underpins band hybridization at the CDW transition across the entire alloy series. Indeed, we note that a similar band flattening has been recently observed in the (2 × 2) CDW phase of ML-ZrTe_2_, where the flattening of the backfolded valence band top has been ascribed as the signature of an excitonic condensation occurring at low temperatures.^25,26^ In our model, however, the band top flattening observed in the 2D alloy can be explained by considering a simple hybridization between the conduction and valence states allowed by the (2 × 2) periodic lattice distortion, and irrespective of its microscopic driving mechanism. Thus, we conclude that spectral signatures like this do not, by themselves, allow us to draw any conclusion about the nature of the coupling.

**Figure 3:**
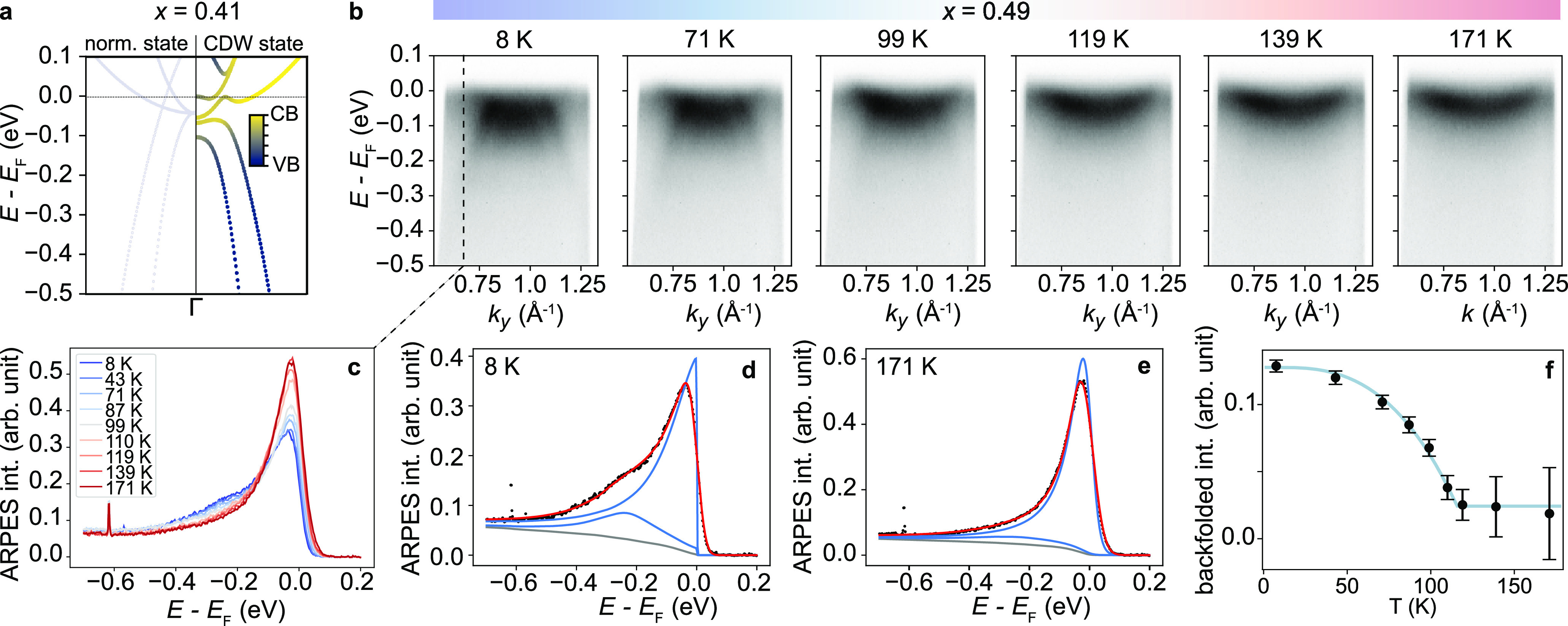
(a) Minimal model simulation of the CDW hybridization in an alloy film with *x* = 0.41 resulting in an almost flat valence band top, similar to what was observed experimentally using ARPES. The valence band (VB) and conduction band (CB) characters of the hybridized dispersions are indicated in dark blue and yellow, respectively. (b) Disappearance of the backfolded spectral weight upon warming a film with *x* = 0.49. (c) Energy distribution curves (EDC) taken at *k*_*y*_ = 0.67 Å^–1^ used to track the evolution of the backfolded intensity in temperature. The peak at around −0.62 eV is an artifact caused by a faulty pixel in the ARPES detector, which, however, does not affect our fitting analysis. (d ,e) Fitting of the high- and low-temperature EDCs in (c). (f) Backfolded spectral weight intensity extracted by fitting the EDCs in (c) at different temperatures showing the characteristic second-order phase transition behavior of the CDW instability with *T*_c_ = 117 ± 5 K.

Nonetheless, since the backfolded intensity follows the amplitude of the (2 × 2) periodic potential and hence the order parameter of the CDW phase, our measurements can be used to estimate how *T*_c_ varies across the alloy series. Figure 3b shows the temperature-dependent ARPES spectrum measured at M for a sample with a composition *x* = 0.49. With increasing temperature, the backfolded spectral weight vanishes, indicating a melting of the CDW order. This temperature evolution can be tracked by extracting energy distribution curves (EDCs) at *k*_*y*_ = 0.67 Å^–1^, as shown in Figure 3c, where the backfolded weight of the outer valence band forms a shoulder at *E* – *E*_F_ ≈ −0.3 eV, whose intensity is suppressed at higher temperatures. The intensity of the backfolded spectral weight was extracted by fitting EDCs at different temperatures with two Lorentzian peaks, a Shirley background, and a Fermi cutoff as shown in Figure 3d,e for *T* = 8 K and *T* = 171 K. The fit function was convoluted with a Gaussian having a fwhm of 17 meV to take into consideration the energy resolution. The ratio between the intensity of the backfolded outer valence band normalized over the intensity of the conduction band (see Figure 3f) shows the typical second-order phase transition scaling behavior as for the CDW transition in ML-TiSe_2_ and ML-TiTe_2_.^4,5,20,21^

We estimate the CDW onset temperature, *T*_c_, by fitting the backfolded valence band intensity with a BCS-like function
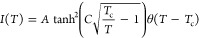
2where *A* and *C* are two free parameters. For the fit shown in Figure 3f, we find a CDW transition temperature of 117 ± 5 K. This is only slightly higher than the established *T*_c_ of 110 K for ML-TiTe_2_,^21^ despite being already around 50% Se-substituted. We apply the same fitting methodology to track the evolution of *T*_c_ across the alloy series. (For compositions where the electron and inner hole pockets are not overlapping (*x* < 0.41) we used the same fitting procedure but for EDCs extracted at M.) We show in Figure 4a the corresponding fitted temperature-dependent intensity of the backfolded bands. This shows a roughly monotonic decrease in the temperature onset of the spectral weight of the backfolded bands with increasing Te content. Despite some variations in sharpness (possibly due to variations in the alloy disorder, see Figure S2 in the Supporting Information) the temperature-dependent onset of spectral weight is well described from fits to eq 2, allowing us to extract the composition-dependent *T*_c_ as shown in Figure 4b. This establishes, for the first time, the experimental phase diagram of the (2 × 2) CDW phase along the entire alloy series of ML-TiTe_2*x*_Se_2(1–*x*)_. We find that as soon as Se is substituted with Te, the critical temperature starts to decrease monotonically up to ∼110 K for *x* ≈ 0.6. For more Te-rich compositions, the critical temperature remains almost constant at ∼110 K up to the pure telluride limit, reflecting a strong dichotomy in the behavior of Te-rich and Te-poor ML-TiTe_2*x*_Se_2(1–*x*)_ alloys.

**Figure 4:**
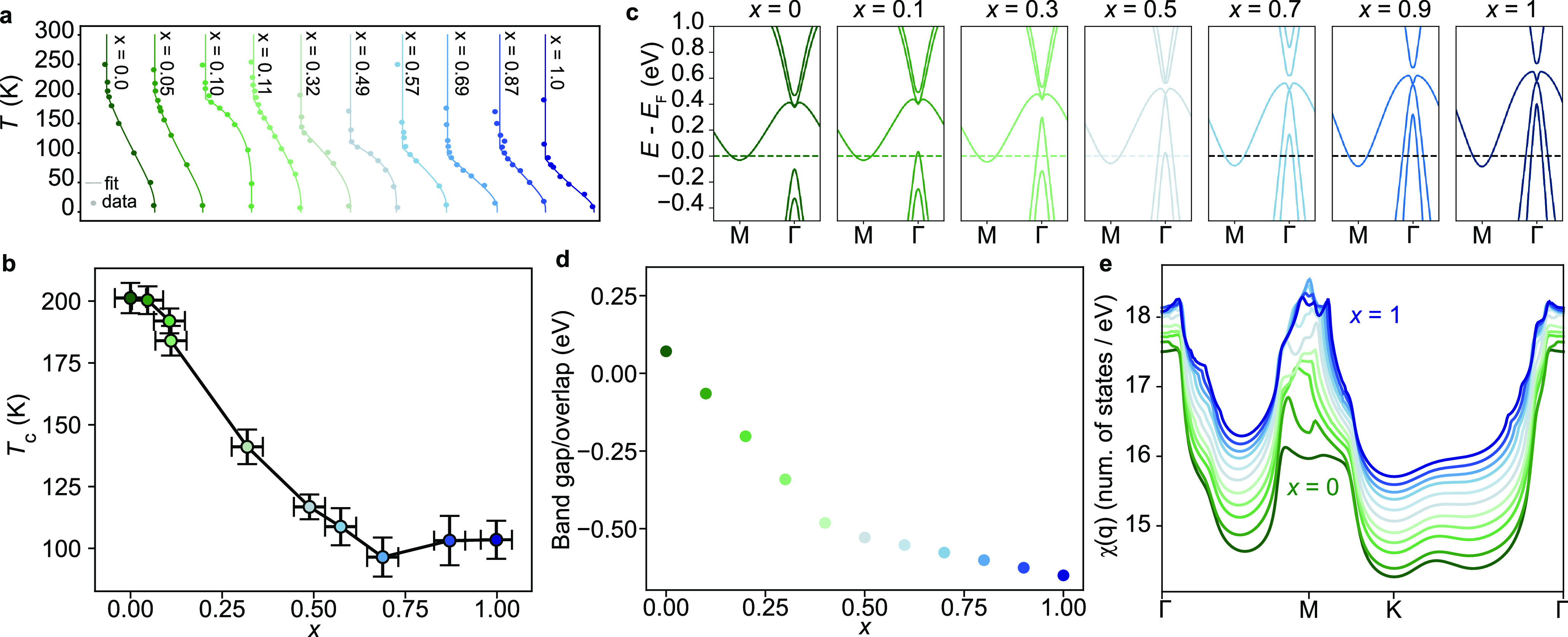
(a) Backfolded intensity vs temperature for ML-TiTe_2*x*_Se_2(1–*x*)_ films with different composition *x* fitted with the BCS-like function in eq 2. (b) Phase diagram of the (2 × 2) CDW phase versus the alloy composition. The sample at *x* = 0.87 was measured at the Bloch beamline of MAX IV Laboratory after being decapped following the procedure outlined in the Supporting Information. (c) Tight-binding calculations for alloys with different compositions from which we extracted the band gap plotted in (d) and the electronic susceptibility, χ(*q*), calculated at *T* = 50 K in (e).

We show below that this behavior closely follows the semiconductor-semimetal crossover of the alloy’s normal state. We calculate the normal state band structure of the 2D alloys using tight-binding models of the form which have previously been shown to well describe the normal state electronic structure of the end members, ML-TiSe_2_ and ML-TiTe_2_.^5.13,21^ Slater–Koster parameters for the alloys were obtained by linear interpolation from the pure telluride and selenide compounds, while the Fermi level was adjusted to match the conduction band minimum observed in our ARPES measurements. Figure 4c shows the calculated band dispersion along the Γ–M path, reproducing the semiconductor to semimetal transition observed by using ARPES (Figure 2e). In Figure 4d we plot the calculated band overlap between the topmost hole band at Γ and the conduction band at M. This shows a relatively fast decrease for *x* < 0.4 while it varies much more slowly for further increase in Te content, a trend remarkably similar to that for *T*_c_. We note that these calculations slightly underestimate the composition *x* at which the gap closing transition occurs, compared to our experimental data in Figure 3. This likely reflects a deviation from linearity of the composition dependence of the tight binding parameters, and including the resulting band gap bowing leads to the same qualitative conclusions (see also Figure S3 in the Supporting Information) as for the linear dependence shown here.

Past work has also shown a monotonic decreasing of *T*_c_ with *increasing* band gap away from the TiSe_2_ end member of bulk TiSe_2*x*_S_2(1–*x*)_ alloys, where the lattice instability is completely suppressed for composition *x* > 0.5.^3,27^ Taken together with our results exploring the evolution into the semimetallic state, we note that this yields an evolution of the CDW instability that is qualitatively similar to the generic phase diagram expected for an excitonic insulator, which is stable only close to the zero-gap point.^28^ Nonetheless, the persistence of a CDW state to TiTe_2_ cannot seemingly be attributed to excitonic correlations, where the increased number of free-charge carriers at the Fermi level is expected to enhance the electronic screening environment inside the 2D crystal. Such a change in electronic screening along the alloy series is evident as an overall increase in the electronic susceptibility, χ, calculated within the Lindhard approximation from our tight-binding band structures, as shown in Figure 4e. Notably, the larger χ(*q* = 0) (i.e., at Γ) in ML-TiTe_2_ (dark blue) points toward a larger electronic screening that is expected to significantly renormalize any excitonic interaction present in ML-TiSe_2_, rapidly suppressing any contribution that this has to boosting *T*_c_. Moreover, rapid changes in the susceptibility are evident with increasing Te concentration up to *x* ≈ 0.5, while only quantitative rather than qualitative variations are obtained for the more Te-rich alloys where the changes are significantly more gradual.

The above considerations are entirely consistent with our qualitative trends observed experimentally, with a rapid decrease in *T*_c_ up until *x* ≈ 0.5, and only subtle changes observed for further increase in Te content. Interestingly, however, we find that the susceptibility for the Te-rich alloys exhibits a more pronounced and narrow peak around M as compared to the selenide-rich alloys, indicating a stronger propensity toward a Peierls-like instability for films with *x* > 0.5. This trend is in contrast to our observation of decreased *T*_c_ for the Te-rich alloys, pointing to the presence of an additional excitonic contribution that cooperatively boosts *T*_c_ in the selenide-rich alloys. More detailed simulations are needed to fully capture the microscopic mechanisms underpinning the phase diagram in Figure 4b, and we hope that our results will serve as motivation for such studies, for example, using first-principles approaches. Nonetheless, our calculations already suggest that controlling the Te content in ML-TiTe_2*x*_Se_2(1–*x*)_ alloys allows tuning the interplay between two mechanisms: a Peierls-like instability, which becomes stronger for the Te-rich compounds, and an excitonic-like coupling, which instead enhances the CDW interaction for the Se-rich alloys where the band gap is close to zero. Our study points to a delicate interplay between these two mechanisms in driving the controversial CDW instabilities in Ti-chalcogenide compounds, leading to the rich phase diagram observed in Figure 4b.

Our report of the first growth of ML-Ti(Se,Te)_2_ alloys establishes this as a model system for harnessing control of the microscopic driving mechanisms of their collective states by the controlled engineering of the normal-state band structure. Our synthesis approach can be readily adopted to other monolayer TMDs, opening powerful routes for tuning their band gaps and improving the versatility of these materials in future electronic and optoelectronic technologies. Here, we have shown that the band gap closure in the Ti-based TMD series promotes a larger Fermi surface where a Peierls-like instability, driven by a peak in the electronic susceptibility, is more likely to occur, but where the larger number of free carriers at the Fermi level also enhances the internal screening, suppressing exciton formation. Our results will thus serve as crucial input to calculations regarding the putative excitonic insulator phase in this and related compounds.

## Data Availability

The research data supporting this publication can be accessed at 10.17630/1b7903b4-d31f-40b3-aa44-5b710570672d.^29^

## Supporting Information Available

Additional information regarding the compositional homogeneity of the alloy films, fits of the BCS-like function, and effects of band gap bowing as simulated via tight-binding calculations (PDF)
