# Supplementary material for: Controlling the Charge Density Wave Transition in Single-Layer TiTe2xSe2(1–x) Alloys by Band Gap Engineering
Source: Nano Lett. 2023 Dec 20;24(1):215–21. doi: 10.1021/acs.nanolett.3c03776 (PMC10786161; doi:10.1021/acs.nanolett.3c03776)
Supplement: Supplementary file 1 — nl3c03776_si_001.pdf [file nl3c03776_si_001.pdf]

Controlling the charge density wave transition in single-layer $TiTe_{2x}Se_{2(1-x)}$ alloys by band gap engineering

Tommaso Antonelli,[1,†] Akhil Rajan,[1] Matthew D. Watson,[1] Shoresh Soltani,[1,‡] Joe Houghton,[1] Gesa-Roxanne Siemann,[1] Andela Zivanovic,[1] Chiara Bigi,[1] Brendan Edwards,[1] and Phil D. C. King[1,*]

[1]*SUPA, School of Physics and Astronomy, University of St Andrews, St Andrews KY16 9SS, United Kingdom*

(Dated: December 13, 2023)

I. SPATIALLY-DEPENDENT SYNCHROTRON XPS

The sample in Fig. 4a,b with composition $x = 0.87$ was measured using the BLOCH beamline at MAX IV synchrotron, which hosts a small ($\approx 10 \times 15$ μm^2) beam spot. In order to prevent the degradation of the alloy film during transport, the as-grown sample was capped with a double capping layer of Te and Se deposited in our MBE chamber, following the procedure outlined in [1]. At the synchrotron endstation, the sample was heated up to $\sim 300°$C for one hour to desorb the capping layer and restore the pristine surface (see Fig. 1(a)). An XPS spectrum of the Se $3d$ and Te $4d$ core levels was measured with linearly horizontal polarised light at 100 eV (Fig. 1(b)). The measurement was repeated on a 17×14 grid covering a 2.5 mm x 2 mm wide area of the sample. The composition at each point was calculated as in Eqn. (1) of the main text, resulting in the spatial map shown in Fig. 1(c). From this, we can analyse the compositional distribution of the film on the sample. Notably, the sample shows a small gradient in composition (red arrow) along one of the diagonals of the sample, likely related to a gradient in temperature on the sample during the decapping procedure. The histogram in Fig. 1(d) shows that the average composition of the film is $x = 0.8$ with a FWHM = 0.1 highlighted by the red area. We expect that the as-grown samples analysed in the main manuscript should have a narrower compositional distribution as no decapping process was performed on them.

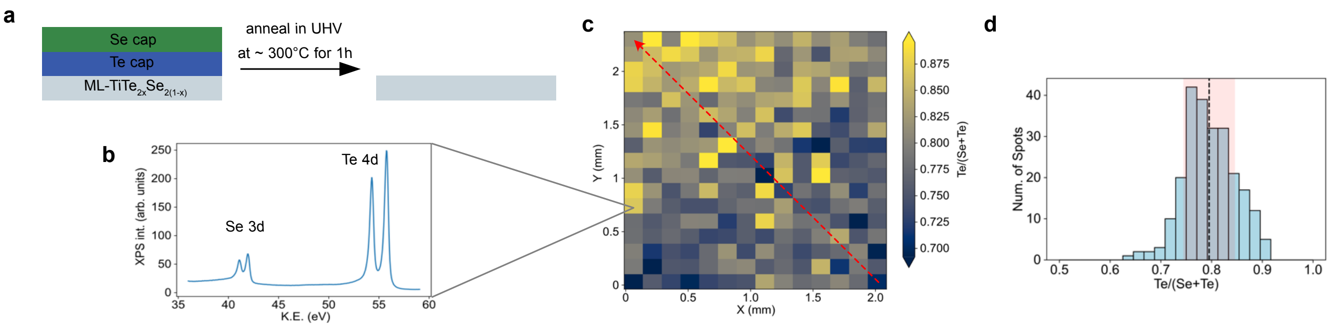

FIG. 1. (a) Schematic of the decapping process performed at the synchrotron end station to restore the pristine surface of the sample. (b) Typical XPS spectrum of the Se $3d$ and Te $4d$ core levels measured on one point of the spatial map. Spatial-dependent alloy composition extracted from XPS spectra showing a clear gradient along one diagonal, probably due to a thermal gradient during the decapping process. (d) Histogram of the local composition showing an average Te content of x=0.8 and FWHM = 0.1.

† tantonell@phys.ethz.ch
‡ Present address: MAX IV Laboratory, Lund University, SE-22100 Lund, Sweden
* philip.king@st-andrews.ac.uk

II. SMOOTHENSS OF THE CDW TRANSITION VS COMPOSITION

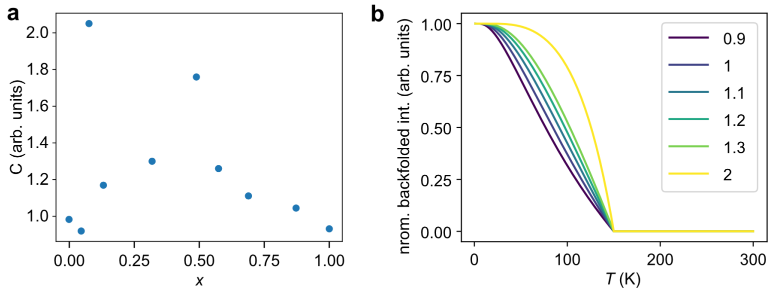

FIG. 2. (a) Variation over the alloy composition of the fitted parameter C in the BCS-like fit function in Eqn. 2 of the main text. This parameter governs the derivative near T_c of the fit function and shows a non-monotonic modulation, in general increasing for compositions close to $x \sim 0.5$, with some scatter on the obtained values. This trend suggests that the CDW transition becomes less "smooth" (higher C, see (b)) for compositions where the intrinsic disorder in the 2D alloy crystal is higher, typically those approaching $x = 0.5$. (b) Simulation of the above-mentioned fit function plotted for different values of C and with fixed T_c. The onset of the transition is not affected by the parameter C since it is dictated by the Heaviside function in Eqn. 2 of the main text. We can therefore get a robust measure of T_c from fits to this function, even though the sharpenss of the onset of backfolded spectral weight varies across the alloy series.

III. BAND-GAP BOWING EFFECT

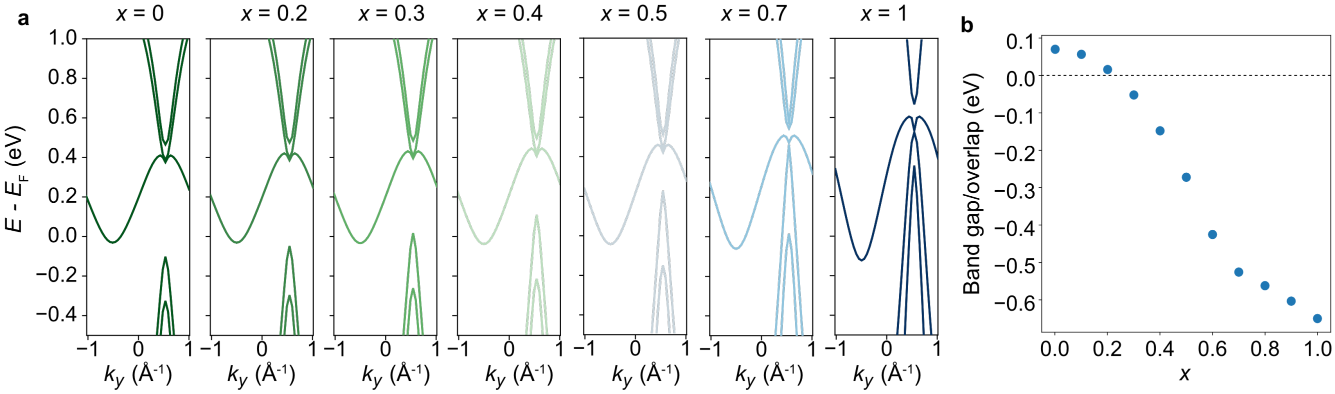

FIG. 3. (a) Band structure simulations at different alloy compositions x using a quadratic dependence of the Slater-Koster parameters, in comparison to the linear variation considered in Fig. 4 of the main text. (b) This leads to a finite bowing in the band gap as a function of alloy composition. Apart from slightly varying the relative compositions at which the band gap closes and where both of the spin-orbit split valence bands cross the chemical potential, the new band structures are qualitatively the same as the ones presented in the main text. Therefore, we conclude that the qualitative correlation between the CDW phase diagram and the modulation of the band gap along the ally series is robust to second-order corrections to our calculations.

IV. REFERENCES

[1] T. Antonelli, W. Rahim, M. D. Watson, A. Rajan, O. J. Clark, A. Danilenko, K. Underwood, I. Marković, E. Abarca-Morales, S. R. Kavanagh, *et al.*, Orbital-selective band hybridisation at the charge density wave transition in monolayer $TiTe_2$, npj Quantum Materials **7**, 98 (2022).
